# Supplementary material for: Germline-like TCR-α chains shared between autoreactive T cells in blood and pancreas
Source: Nat Commun. 2024 Jun 13;15:4971. doi: 10.1038/s41467-024-48833-w (PMC11176301; doi:10.1038/s41467-024-48833-w)
Supplement: Supplementary file 1 — Supplementary Information [file 41467_2024_48833_MOESM1_ESM.pdf]

Germline-like TCR-α chains shared between autoreactive T cells in blood and pancreas

Peter S. Linsley [1†], Maki Nakayama [2], Elisa Balmas[1], Janice Chen[1], Fariba Barahmand-pour-Whitman [1], Shubham Bansal [1], Ty Bottorff [1], Elisavet Serti [3], Cate Speake [1], Alberto Pugliese [4], and Karen Cerosaletti [1].

[1] Benaroya Research Institute at Virginia Mason, Seattle, Washington, USA.

[2] Barbara Davis Center for Childhood Diabetes, Department of Immunology and Microbiology, University of Colorado School of Medicine, Aurora, CO, USA.

[3]Immune Tolerance Network, Bethesda, Maryland, USA.

[4] Department of Diabetes Immunology & The Wanek Family Project for Type 1 Diabetes, Arthur Riggs Diabetes & Metabolism Research Institute, City of Hope, Duarte, CA, USA

Supplementary Data

Supplementary Data 1. Characteristics of study participants.

Supplementary Data 2. Compiled and filtered TCR sequences used in this study.

Supplementary Data 3. Peptide contacts with IAR TCR sequence features.

Supplementary Data 4. Overlapping islet peptide libraries.

Supplementary Data 5. Flow cytometry antibodies.

Supplementary Data 6. DNA sequence encoding P196-1 TCR

Supplementary Figures

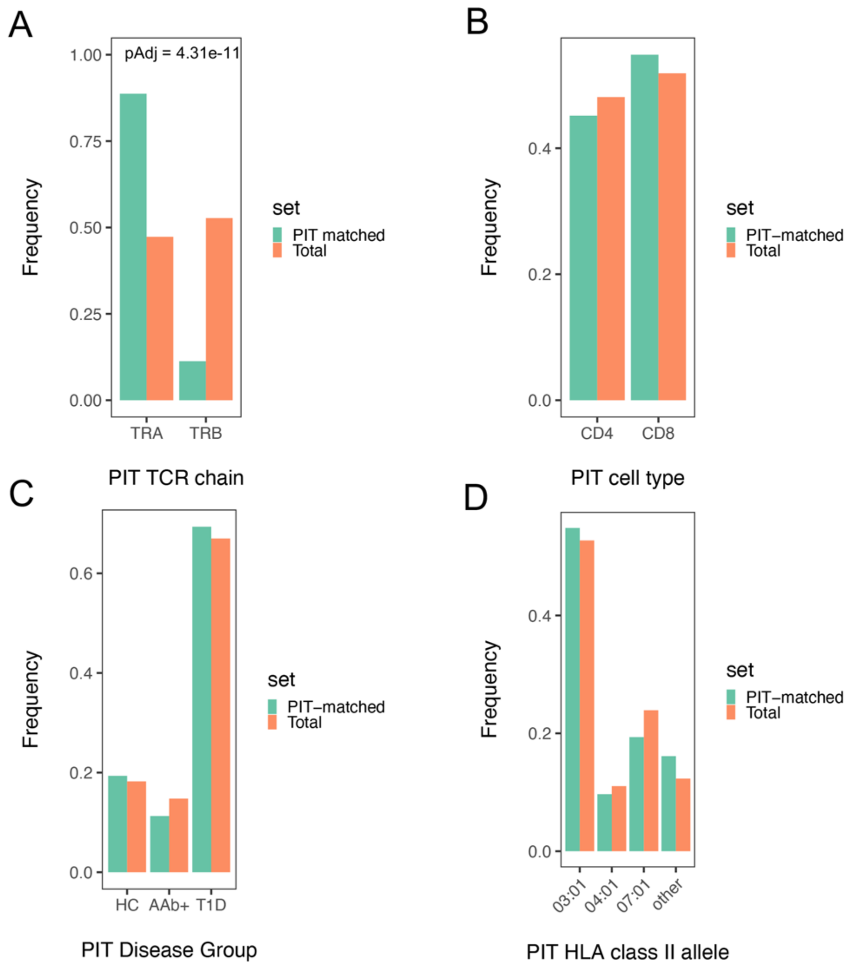

Supplementary Figure 1. Characteristics of IAR matching versus total PIT TCRs. A) TCR chain types in perfectly IAR-matching *Cohort 1* (n = 55 *TRA* and 7 *TRB*) and total unique PIT TCRs (n = 9, 757 combined *TRA* and *TRB*, **Table 2**). B) Cell types in IAR-matching (n = 62) and total unique PIT TCRs (total n = 4,695 and 5,063 CD4 and CD8, respectively). C) Patient disease groups of donors in IAR-matching (n = 62) and total unique PIT TCRs (total n = 1,779 HC, 1,442 AAb+ and 6,536 T1D). D) HLA class II alleles of in donors of IAR-matching (n = 62) and total unique PIT TCRs (total n = 5,144 03:01, 1,077 04:01, 2,334 07:01 and 1,202 alleles other than 03:01, 04:01 and 07:01). Differences between groups were assessed using Fisher's exact test.

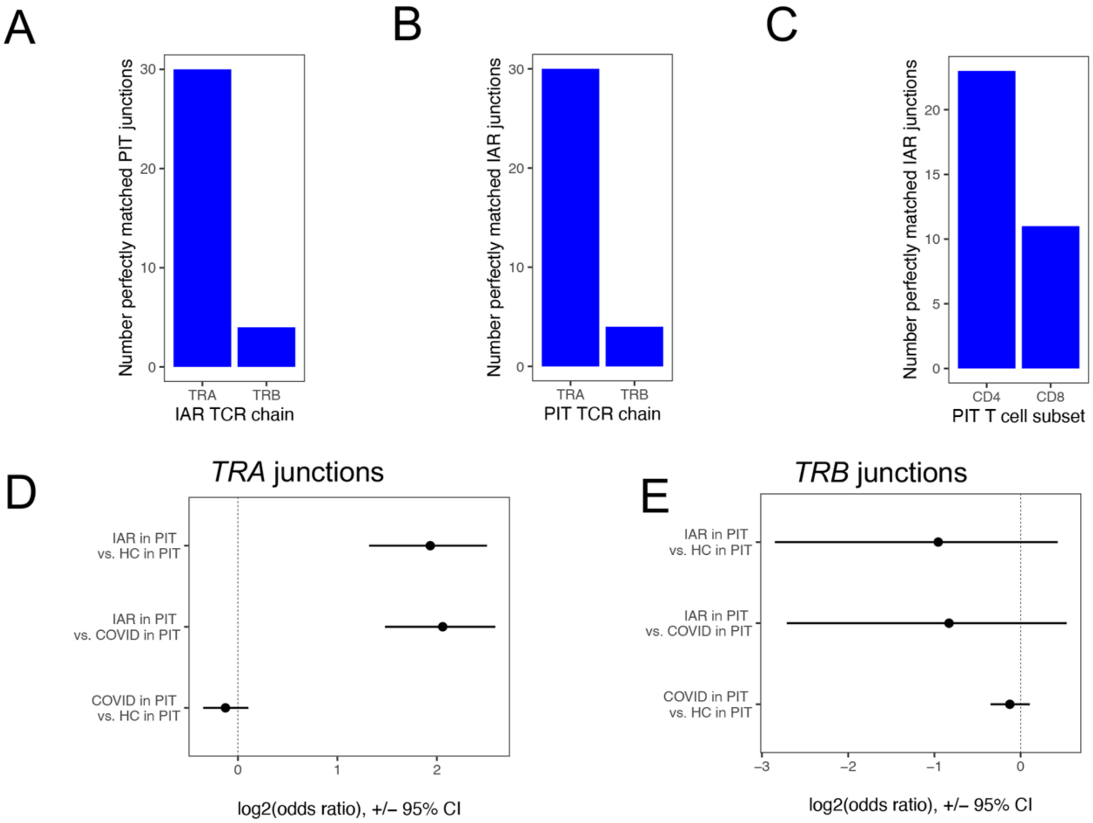

Supplementary Figure 2. IAR T and PIT TCR sequence matching in *Cohort 2*. A-G) As in **Figure 1** but using TCRs from *Cohort 2*. A) Perfectly matched IAR *TRA* (n = 30) and *TRB* (n = 4) junctions from *Cohort 2* (**Table 2**) in PIT TCRs (n = 9,757 total unique *TRA* and *TRB* junctions, **Table 2**). B) Perfectly matched PIT *TRA* (n = 30) and *TRB* (n = 4) junctions in IAR TCRs (n = 2,141 total unique *TRA* and *TRB* junctions, **Table 2**). C) Perfectly matched PIT T cell TCR junctions from CD4+ (n = 23) and CD8+ (n = 11) cells in IAR TCRs. D) PIT-matched versus non-matched *TRA* junctions from *Cohort 2* IAR T cell TCRs (n = 30 matched and 1,060 non-matched) compared with *TRA* junctions from unselected HC[20] (185 matched and 24,968 non-matched junctions); and COVID-19 patients[20] (1,341 matched and 197,412 non-matched junctions). Log_2 odds ratios were calculated by Fischer's exact test. Error bars, 95% confidence intervals. Dotted vertical line, equivalency line. E) As in D but using *TRB* junctions (n = 4 matched and 1,047 non-matched).

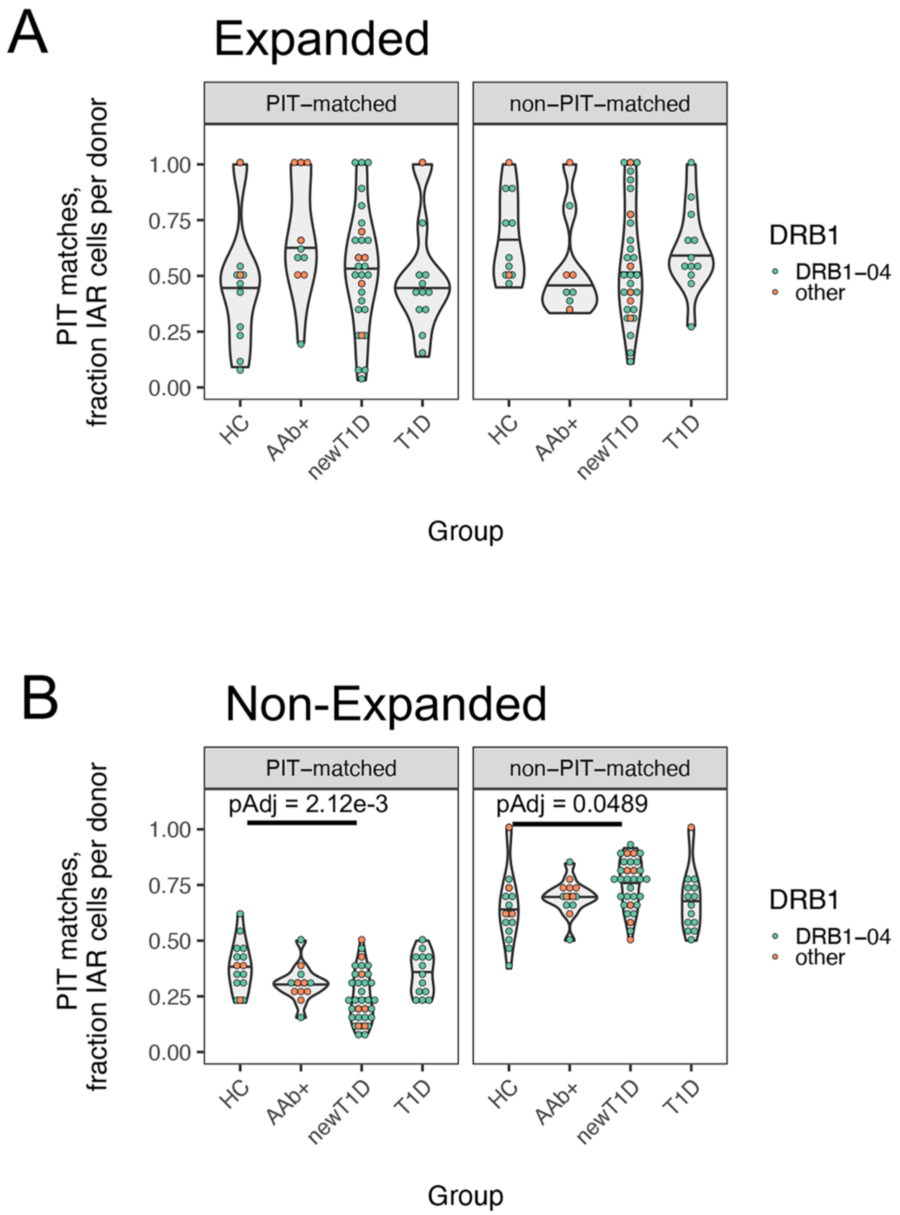

Supplementary Figure 3. Fractions of PIT-matched and non-matched *TRA* junctions by disease group and TCR expansion. Tabulated frequencies of PIT-matched and non-matched *TRA* junction numbers by group in combined *Cohorts 1 + 2* as in **Figure 1F** but analyzed at the donor level (**Table 1, Supplementary Data 1**). Differences were assessed using Wilcoxon signed-rank test. Each dot represents a value for an individual donor, colored by HLA-DRB1 allele. The width of the violins represent frequency; horizontal lines within the violins represent median values.

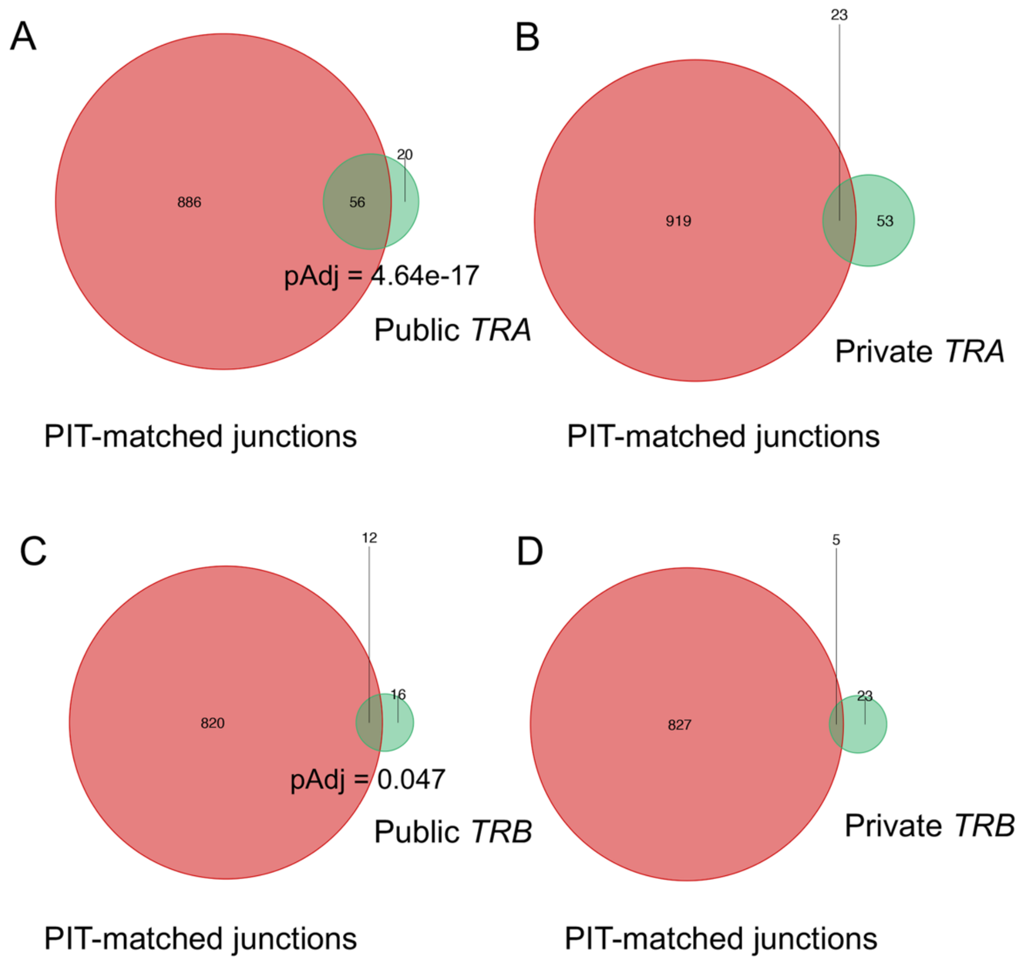

Supplementary Figure 4. Enrichment of public sequences in PIT-matched *TRA* but not *TRB* junctions. A) Intersections of unique IAR PIT-matching *TRA* junctions (N = 942) in combined *Cohorts 1 + 2*, with unique public IAR *TRA* junctions (n = 76). B) Intersections of IAR PIT-matching *TRA* junctions, with private IAR *TRA* junctions, down sampled (Methods) to equal numbers as public *TRA* junctions (n = 76). C) Intersections of unique IAR PIT-matching *TRB* junctions (n = 832), with public IAR *TRB* junctions (n = 28). D) Intersections of unique IAR PIT-matching *TRB* junctions (N = 832), and private IAR *TRB* junctions, down sampled to equal numbers as public *TRB* junctions (n = 28). "Universe" values for hypergeometric p-value calculations were n= 3,264 and 3,187 for total unique *TRA* and *TRB* junctions, respectively (**Table 2**). Differences were assessed using Fisher's exact test.

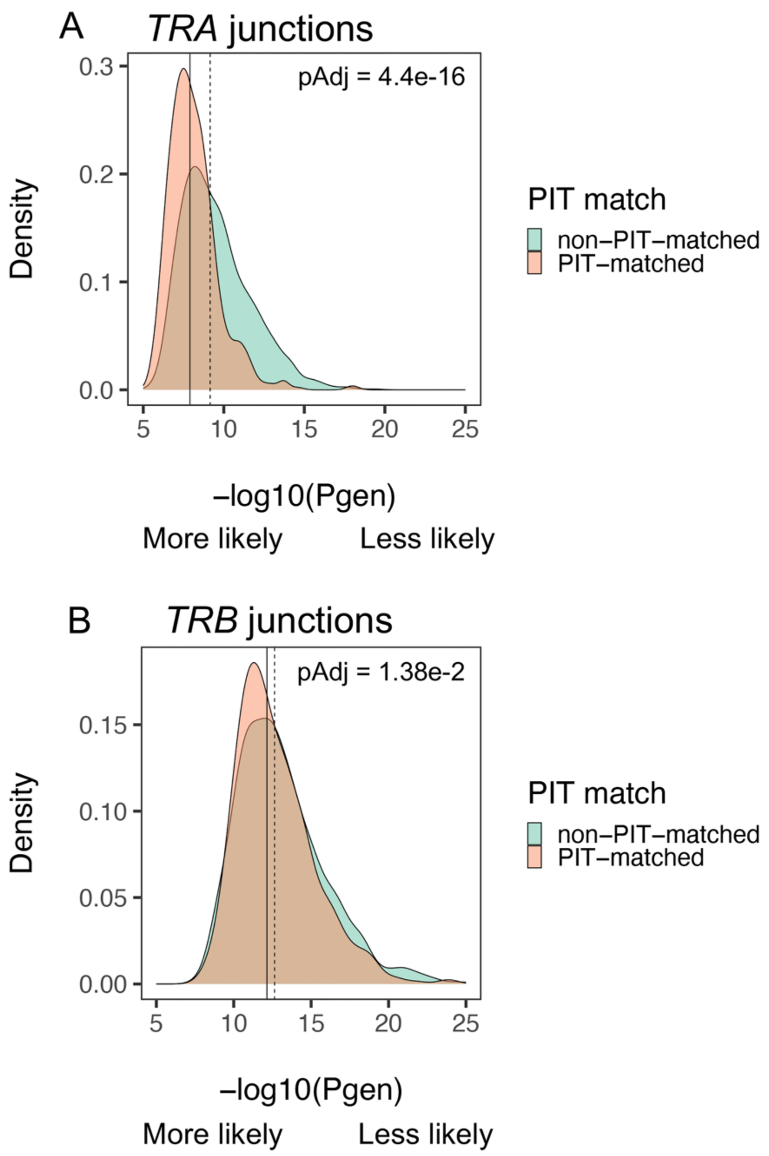

Supplementary Figure 5. PIT-matched junctions have higher generation probability relative to PIT non-matched junctions. Shown are distributions of generation probabilities (*Pgen*)[1] for PIT-matched and non-PIT-matched *TRA* (n = 942 and n = 2,322, respectively) (A) and *TRB* (B) (n = 832 and n = 2,355, respectively) junctions from combined *Cohorts 1 + 2*. Differences were assessed using Kolmogorov-Smirnov test. Solid vertical line, median value from PIT-matched junctions; dashed vertical line, median value from non-PIT-matched junctions.

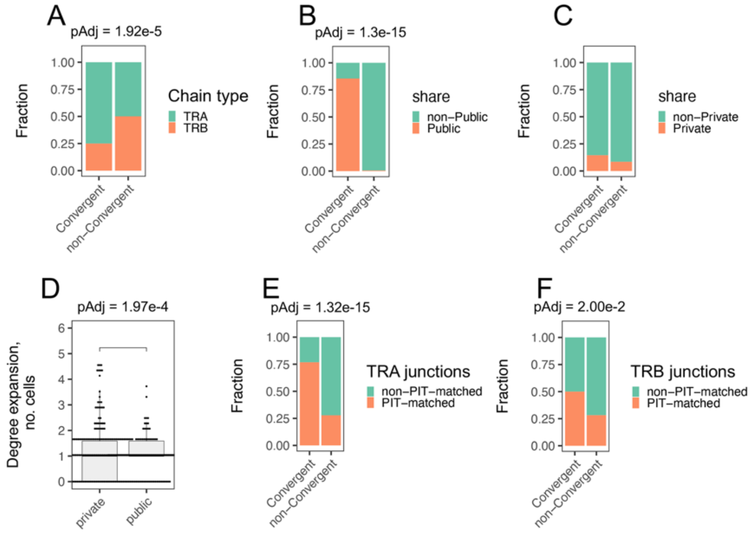

Supplementary Figure 6. Convergence of public TRA junction nucleotide sequences. A) Frequencies of convergent *TRA* and *TRB* junctions. Convergent, number of junction AA sequences encoded by >one nt sequence. There were 90 total convergent junctions (69 *TRA* and 21 *TRB*), and 6,361 non-convergent junctions (~1.4% convergent). Differences were assessed using Fisher's exact test. B) Frequencies of convergent sequences in public and non-public *TRA* junctions. Public, junction having identical AA sequences in >one donor. There were 76 public and 3,188 non-public *TRA* junctions. Differences were assessed using Fisher's exact test. C) Frequencies of convergent sequences in private and non-private *TRA* junctions. Private, junction having identical AA sequences in >1 cell from a single donor only. There were 281 private and 2,983 non-private *TRA* junctions. Differences were assessed using Fisher's exact test. D) Expansion in public and private *TRA* junctions. Expansion, identical junction AA sequences found in >1 cell from one or more donors. There were 357 expanded and 2,907 non-expanded *TRA* junctions. E) Frequencies of PIT-matched sequences in convergent *TRA* junctions. There were 942 PIT-matched and 2,322 non-PIT-matched *TRA* junctions. Differences were assessed using Wilcoxon signed rank test. F). Frequencies of PIT-matched sequences in convergent *TRB* junctions. There were 832 PIT-matched and 2,355 non-PIT-matched *TRB* junctions. Differences were assessed using Fisher's exact test.

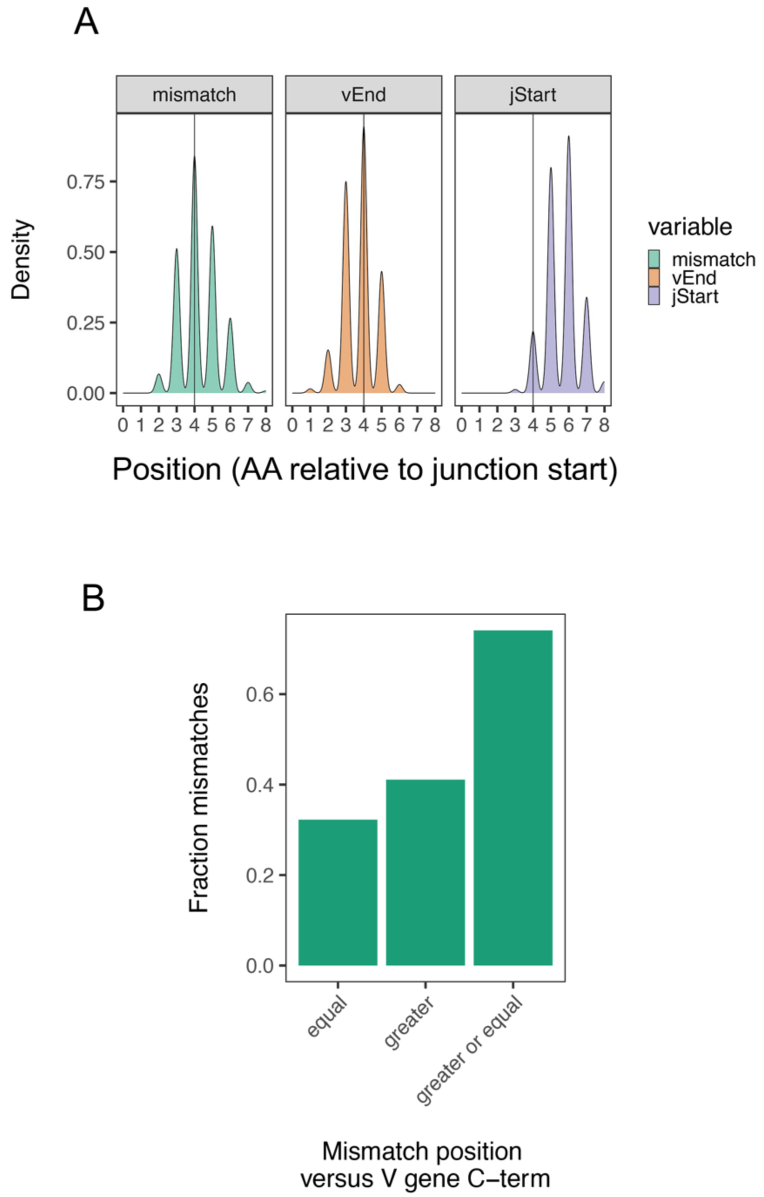

Supplementary Figure 7. Mismatches between IAR and PIT *TRA* junctions map to templated and non-templated regions. A) Aggregated distribution of AA sequence positions of PIT mismatches between IAR *TRA* and PIT junctions (n = 927 unique IAR TRA junctions with 1 mismatch to a PIT junction). N terminal AA residues from all IAR *TRA* junctions were assigned position = 0. The distribution of positions of observed mismatches was then compared to the C-terminal ends of *V genes* (vEnd) and the N-terminal ends of *J genes* (jStart), as delineated by IMGT/HighV-QUES [2]. Vertical lines, median position of all PIT mismatches. B) Positions of single

PIT mismatches (n = 927) relative to the C-terminal end of the *V gene* in each individual *TRA* junction.

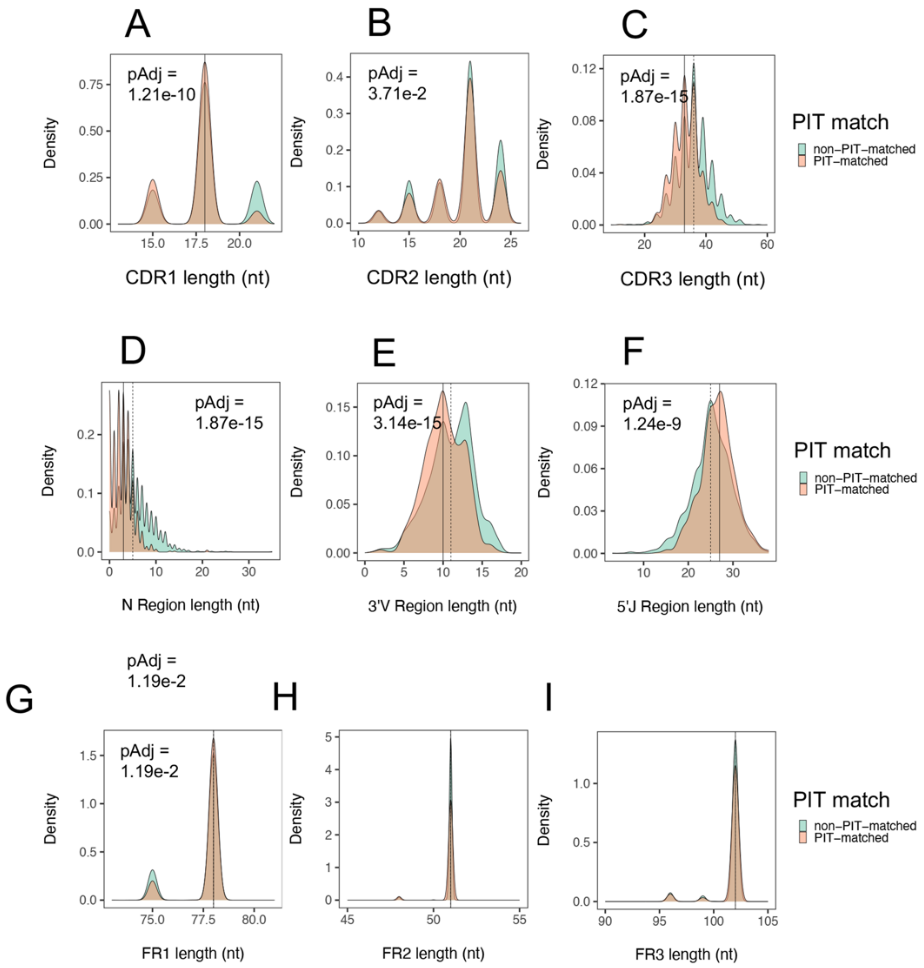

Supplementary Figure 8. Length differences between PIT-matched and non-matched *TRA* junctions in peptide-contacting regions. Distributions of sequence lengths (in nt) for features of PIT-matched (n = 942 and 832) and non-matched (n = 2,322 and 2,355) *TRA* and *TRB* junctions, respectively, as delineated by IMGT/HighV-QUEST [2] from combined *Cohorts 1*+ *2*. A) CDR1; B) CDR2; C) CDR3; D) N region; E) 3' end of V gene (3' V); F) 5'end of the J region (5'J); G-I) Framework regions FR1, FR2 and FR3, respectively. Differences in values from PIT-matched and non-matched chains were assessed using Kolmogorov-Smirnov test. Solid vertical line, median value from PIT-matched junctions; dashed vertical line, median value from PIT-non-matched junctions.

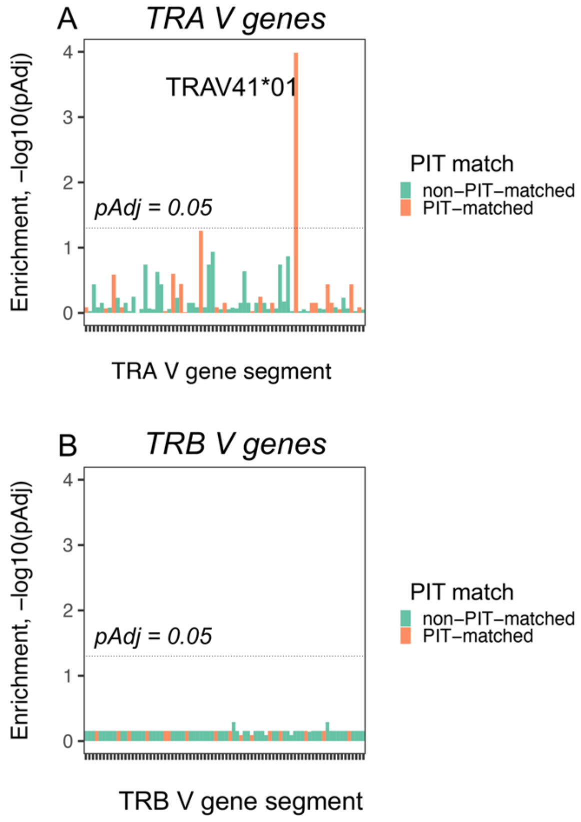

Supplementary Figure 9. *TRA* V genes enriched in PIT- matched versus non-PIT-matched TCRs. We enumerated numbers of PIT-matches and -non-matches for each *TRA* and *TRB* V gene found in IAR T cells using IMGT/HighV-QUEST [2] (n = 71 *TRA V* genes, 78 *TRB V genes*, **Supplementary Data 2**). Shown is a bar plot of -log10 FDR-adjusted (pAdj) values for differences between abundance for each V gene in PIT-matched and non-matched TCR chains, determined by Fisher's exact test. Dotted line, pAdj = 0.05.

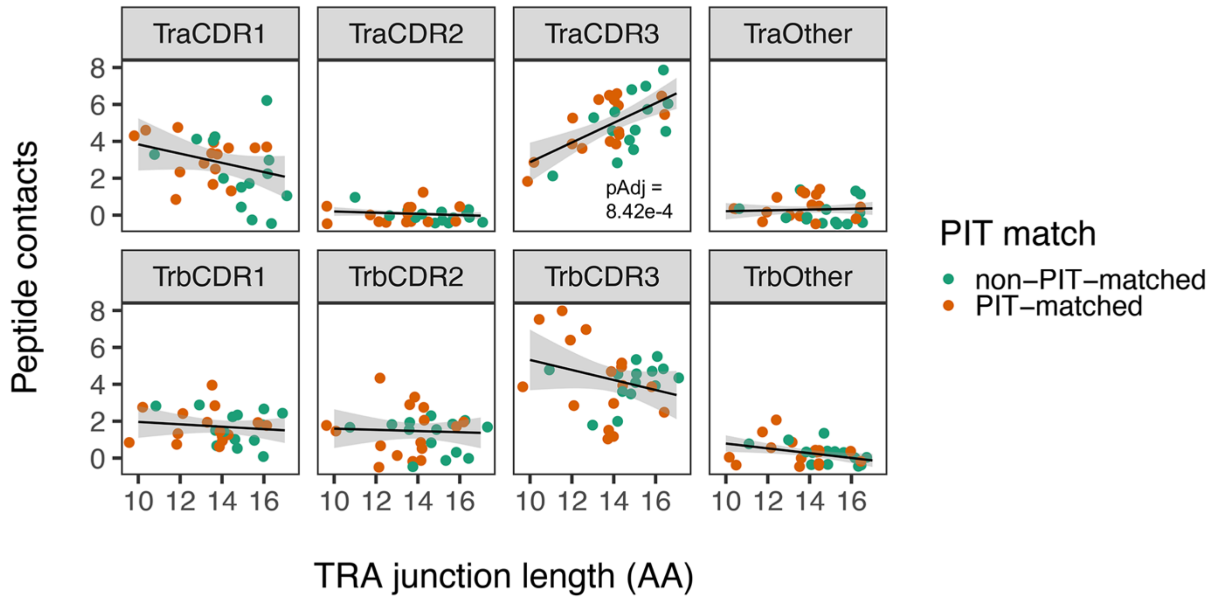

Supplementary Figure 10. Distribution of peptide contact regions in PIT-matched and PIT-non-matched TCR sequence features. We identified predicted peptide contact residues from molecular models of PIT-matched and PIT-non-matched TCRs and mapped these to various sequence features. Plotted are numbers of contact residues in each TCR chain sequence by subject feature plotted versus *TRA* junction length. Difference in slopes of best fit lines from zero were calculated using linear modeling.

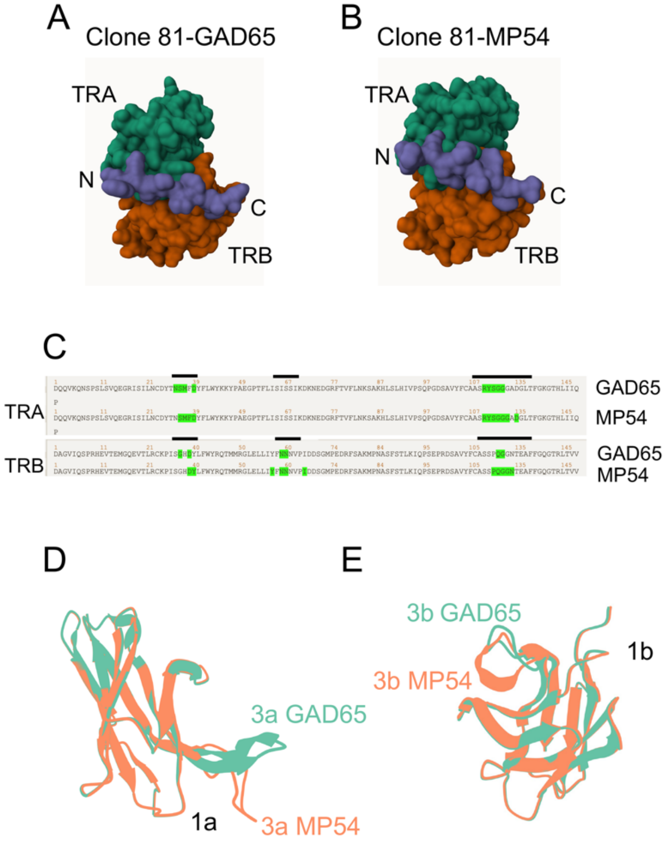

Supplementary Figure 11. Molecular modeling of a cross-reactive PIT-matched TCR with two different peptides. Molecular models of Clone 81 TCR with GAD65 377-396 and MP74 97-116 MHC class II DRA*0101/DRB1*0401 complexes. N and C, peptide amino and carboxy termini, respectively. A-B). Predicted overall topological similarity of tripartite models of Clone 81 TCRs with GAD65 377-396 and MP74 97-116 MHC class II complexes. The viewing plane is from the interface with the MHC molecules, which have been removed from the representation for clarity. C) Green font, peptide contact residues; bars, CDR 1, CDR2 and CDR3 regions, from left to right. There were no predicted contact residues in the *TRA* CDR2 region. D-E) Alignment of predicted structures for the Clone 81 *TRA* (D) and TRB (E) chains. Green, sequences for MP54 peptide models; orange, GAD65 peptide models. Labels indicate the CDR loops of the respective chains. CDR3 regions, which are labeled because they show the greatest divergence between the two models. Outside the CDR loops, the structures were mostly superimposed and may not be separately visible.

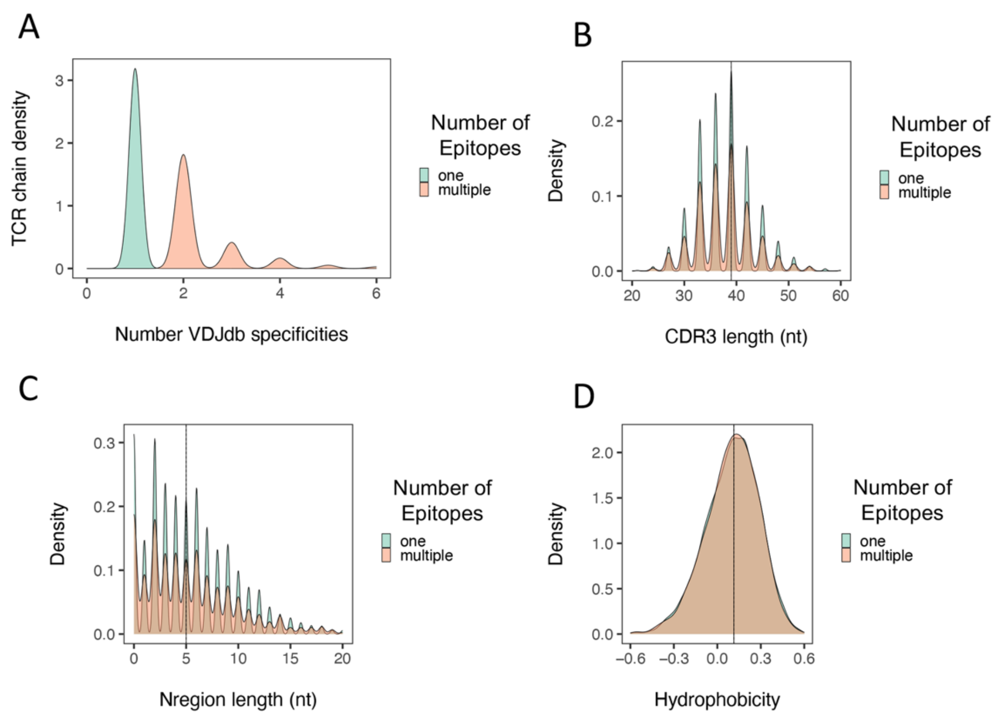

Supplementary Figure 12. *TRB* junctions from cross-reactive TCRs in the *VDJdb* database were not shorter and did not encode more hydrophobic peptide sequences than junctions from single specificity TCRs. A) Density of *TRB* chains from TCRs with one versus multiple specificities in *VDJdb*. There were N = 19,508 unique *TRB* chains from TCRs with single specificity and N = 1,292 with multiple specificities. B) Distribution of *TRB* CDR3 nt lengths from TCRs with one versus multiple specificities; C) Distribution of *TRB* N region nt lengths. D) Distribution of *TRB* junction AA hydrophobicity. Solid vertical lines, median values from TCRs with multiple specificities; Dashed vertical lines, median values in *TRB* regions from TCRs with single reported specificities. Differences in distributions were assessed using Kolmogorov-Smirnov tests.

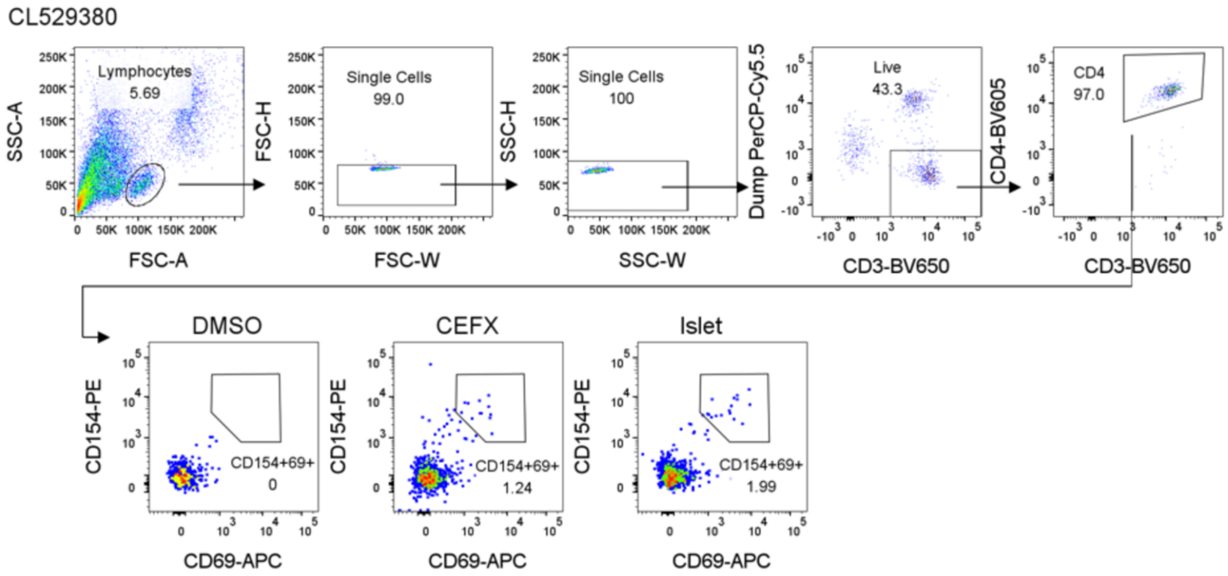

Supplementary Figure 13. Gating strategy for flow cytometry identification of IAR CD4+ T cells for single cell sorting from sample, CL529380. PBMC were stimulated with DMSO (vehicle), foreign antigen (CEFX, positive control), or a pool of class II optimized peptides from islet proteins after overnight culture. Cells were gated as lymphocytes →single cells (X2) →Live, CD3+ cells (dump included Viaprobe, anti-CD8, -CD19, -CD14, -CD56, -CD161, and -Vα24-Jα18 to remove iNKT cells) →CD4+ T cells. Activated cells within the CD4+ T cell population were identified as CD154+CD69+.

References.

1. Sethna Z, Elhanati Y, Callan CG, Walczak AM, Mora T. OLGA: fast computation of generation probabilities of B- and T-cell receptor amino acid sequences and motifs. Bioinformatics. 2019 Sep 1;35(17):2974–2981. PMCID: PMC6735909

2. Alamyar E, Duroux P, Lefranc MP, Giudicelli V. IMGT(®) tools for the nucleotide analysis of immunoglobulin (IG) and T cell receptor (TR) V-(D)-J repertoires, polymorphisms, and IG mutations: IMGT/V-QUEST and IMGT/HighV-QUEST for NGS. Methods Mol Biol. 2012;882:569–604. PMID: 22665256
