# Supplementary material for: Germline-like TCR-α chains shared between autoreactive T cells in blood and pancreas
Source: Nat Commun. 2024 Jun 13;15:4971. doi: 10.1038/s41467-024-48833-w (PMC11176301; doi:10.1038/s41467-024-48833-w)
Supplement: Supplementary file 3 — Description of Additional Supplementary Files [file 41467_2024_48833_MOESM3_ESM.pdf]

Description of Additional Supplementary Files

File Name: Supplementary Data 1

Description: Charactertistics of study particpants.

File Name: Supplementary Data 2

Description: Compiled and filtered TCR sequences used in this study.

File Name: Supplementary Data 3

Description: Peptide contacts with IAR TCR sequence features.

File Name: Supplementary Data 4

Description: Overlapping islet peptide libraries.

File Name: Supplementary Data 5

Description: Flow cytometry antibodies.

File Name: Supplementary Data 6

Description: DNA sequence encoding P196-1 TCR.
