# Supplementary material for: Germline-like TCR-α chains shared between autoreactive T cells in blood and pancreas
Source: Nat Commun. 2024 Jun 13;15:4971. doi: 10.1038/s41467-024-48833-w (PMC11176301; doi:10.1038/s41467-024-48833-w)
Supplement: Supplementary file 10 — Reporting Summary [file 41467_2024_48833_MOESM10_ESM.pdf]

nature portfolio

Corresponding author(s): Peter Linsley

Last updated by author(s): Apr 16, 2024

Reporting Summary

Nature Portfolio wishes to improve the reproducibility of the work that we publish. This form provides structure for consistency and transparency in reporting. For further information on Nature Portfolio policies, see our Editorial Policies and the Editorial Policy Checklist.

Statistics

For all statistical analyses, confirm that the following items are present in the figure legend, table legend, main text, or Methods section.

n/a	Confirmed	
☐	☒	The exact sample size (*n*) for each experimental group/condition, given as a discrete number and unit of measurement
☐	☒	A statement on whether measurements were taken from distinct samples or whether the same sample was measured repeatedly
☐	☒	The statistical test(s) used AND whether they are one- or two-sided<br>*Only common tests should be described solely by name; describe more complex techniques in the Methods section.*
☐	☒	A description of all covariates tested
☐	☒	A description of any assumptions or corrections, such as tests of normality and adjustment for multiple comparisons
☐	☒	A full description of the statistical parameters including central tendency (e.g. means) or other basic estimates (e.g. regression coefficient) AND variation (e.g. standard deviation) or associated estimates of uncertainty (e.g. confidence intervals)
☐	☒	For null hypothesis testing, the test statistic (e.g. *F*, *t*, *r*) with confidence intervals, effect sizes, degrees of freedom and *P* value noted<br>*Give P values as exact values whenever suitable.*
☒	☐	For Bayesian analysis, information on the choice of priors and Markov chain Monte Carlo settings
☒	☐	For hierarchical and complex designs, identification of the appropriate level for tests and full reporting of outcomes
☒	☐	Estimates of effect sizes (e.g. Cohen's *d*, Pearson's *r*), indicating how they were calculated

Our web collection on statistics for biologists contains articles on many of the points above.

Software and code

Policy information about availability of computer code

Data collection

Data collection is described in the GEO repository under accession numbers, GSE182870 and GSE255400. RNA-sequencing was performed on a Illumina NextSeq. Data processing was as described previously (PMID: PMID: 34806648).

Data analysis

Statistical tests were performed using the R programming language and software environment (v. 4.2.3). Plots were made using ggplot2 (v.3.5.0). Additional R software packages included: stringdist (v. 0.9.10); Stitchr (v. 1.1.3.1); and the following packages:

attached base packages:
[1] stats4 stats graphics grDevices utils datasets methods base

other attached packages:
[1] readxl_1.4.3 scales_1.3.0 stringdist_0.9.12 r2symbols_1.4
[5] ggplot2_3.5.0 psych_2.4.1 reshape2_1.4.4 gdata_3.0.0
[9] plyr_1.8.9 limma_3.54.2 Biostrings_2.66.0 GenomeInfoDb_1.34.9
[13] XVector_0.38.0 IRanges_2.32.0 S4Vectors_0.36.2 BiocGenerics_0.44.0

loaded via a namespace (and not attached):
[1] gtools_3.9.5 tidyselect_1.2.0 lattice_0.22-5
[4] colorspace_2.1-0 vctrs_0.6.5 generics_0.1.3
[7] htmltools_0.5.7 utf8_1.2.4 rlang_1.1.3
[10] pillar_1.9.0 glue_1.7.0 withr_3.0.0
[13] GenomeInfoDbData_1.2.9 lifecycle_1.0.4 stringr_1.5.1

```
[16] zlibbioc_1.44.0     cellranger_1.1.0     munsell_0.5.0
[19] gtable_0.3.4        ragg_1.2.7           labeling_0.4.3
[22] fastmap_1.1.1       parallel_4.2.3       fansi_1.0.6
[25] Rcpp_1.0.12         jsonlite_1.8.8       farver_2.1.1
[28] systemfonts_1.0.5   textshaping_0.3.7    mnormt_2.1.1
[31] digest_0.6.34       stringi_1.8.3        dplyr_1.1.4
[34] grid_4.2.3          cli_3.6.2            tools_4.2.3
[37] bitops_1.0-7        magrittr_2.0.3       RCurl_1.98-1.14
[40] tibble_3.2.1        crayon_1.5.2         pkgconfig_2.0.3
[43] rstudioapi_0.15.0   R6_2.5.1             nlme_3.1-164
[46] compiler_4.2.3
```

For manuscripts utilizing custom algorithms or software that are central to the research but not yet described in published literature, software must be made available to editors and reviewers. We strongly encourage code deposition in a community repository (e.g. GitHub). See the Nature Portfolio guidelines for submitting code & software for further information.

Data

Policy information about availability of data

All manuscripts must include a data availability statement. This statement should provide the following information, where applicable:

- Accession codes, unique identifiers, or web links for publicly available datasets
- A description of any restrictions on data availability
- For clinical datasets or third party data, please ensure that the statement adheres to our policy

The profiles yielding TCR data generated in this study from Cohorts 1 and 2 have been deposited in the GEO repository under accession numbers GSE182870 (https://www.ncbi.nlm.nih.gov/geo/query/acc.cgi?acc=GSE182870) and GSE256481 (https://www.ncbi.nlm.nih.gov/geo/query/acc.cgi?acc=GSE256481). PIT TCR sequences were deposited in the iReceptor repository (Study ID: DOI:10.1073/pnas.2107208118). TCR sequences and models (.pdb files) are also available at at Figshare (Linsley, Peter; Nakayama, Maki; Balmas, Elisa; Chen, Janice; Barahmand-pour, Fariba; Bansal, Shubham; et al. (2024). Self-reactive germline-like TCR alpha chains shared between blood and pancreas. figshare. Dataset. https://doi.org/10.6084/m9.figshare.24309679). Other data files used to generate figures are available at GitHub (https://github.com/BenaroyaResearch/Germline-like-TCR-alpha-chains-shared-between-autoreactive-T-cells-in-blood-and-pancreas).

Research involving human participants, their data, or biological material

Policy information about studies with human participants or human data. See also policy information about sex, gender (identity/presentation), and sexual orientation and race, ethnicity and racism.

Reporting on sex and gender	Since T1D affects individuals of both sexes, subjects for this study were selected without regard to sex.
Reporting on race, ethnicity, or other socially relevant groupings	Race, ethnicity, or other socially relevant groupings were recorded but not used as the basis for subject selection.
Population characteristics	Population characteristics are provided in 'Supplementary Table 1'. Also described there are covariate-relevant population characteristics of the human research participants such as age and sex, and when available, C-peptide and antibody characteristics.
Recruitment	Subjects were recruited independently by several organizations as described in the text.
Ethics oversight	Samples and clinical data that were collected under the auspices of this trial were provided to researchers without containing patient specific information. Oversite for the use of patient samples for these experiments at Benaroya Research Institute was approved under protocol, IRB07109-332..

Note that full information on the approval of the study protocol must also be provided in the manuscript.

Field-specific reporting

Please select the one below that is the best fit for your research. If you are not sure, read the appropriate sections before making your selection.

☒ Life sciences ☐ Behavioural & social sciences ☐ Ecological, evolutionary & environmental sciences

For a reference copy of the document with all sections, see nature.com/documents/nr-reporting-summary-flat.pdf

Life sciences study design

All studies must disclose on these points even when the disclosure is negative.

Sample size	Sample sizes were selected by balancing cost versus power.
Data exclusions	iNKT and MAIT TCRs were previously known to be abundant TCRs that recognize carbohydrate and bacterial metabolites, respectively. As we were interested in islet protein epitopes in the present study, we viewed these TCRs recognizing low molecular weight antigens as confounders and removed them from analysis.

Replication	Cohort 1 and Cohort gave essentially identical results (Figure 1 and Figure S2).
Randomization	Allocation into experimental groups was based on clinical variables.
Blinding	RNA-sequencing and cytometry experiments were performed blinded (i.e., the experimenters did not know the clinical characteristics of samples tested). Though blinded, control and experimental groups were typically run in pairs to randomize effects of technical biases on biological groupings

Reporting for specific materials, systems and methods

We require information from authors about some types of materials, experimental systems and methods used in many studies. Here, indicate whether each material, system or method listed is relevant to your study. If you are not sure if a list item applies to your research, read the appropriate section before selecting a response.

Materials & experimental systems

n/a	Involved in the study	
☐	☒	Antibodies
☒	☐	Eukaryotic cell lines
☒	☐	Palaeontology and archaeology
☒	☐	Animals and other organisms
☐	☒	Clinical data
☒	☐	Dual use research of concern
☒	☐	Plants

Methods

n/a	Involved in the study	
☒	☐	ChIP-seq
☐	☒	Flow cytometry
☒	☐	MRI-based neuroimaging

Antibodies

Antibodies used

Antibodies used are described in Supplemental Table S5 as follows

Florophore	Ab	Clone	Supplier	Catalog number	Titer
FITC	SLAMF6	REA339	Miltenyi	130-131-096	1:200
PerCP-Cy5.5	CD8	SK1	BD Pharmigen	565310	1:100
PerCP-Cy5.5	CD19	HIB19	Biolegend	302202	1:100
PerCP-Cy5.5	CD14	61D3	eBioscience	45-0149-42	1:100
PerCP-Cy5.5	CD56	B159	BD Pharmigen	560842	1:100
PerCP-Cy5.5	iNKT	6B11	Biolegend	342914	1:100
PerCP-Cy5.5	CD161	HP-3G10	Biolegend	339908	1:100
Viaprobe	Live/Dead		BD Pharmigen	555815	1:20
BV421	CCR4	L291H4	Biolegend	359414	1:100
BV510	CCR6	11A9	BD Horizon	563241	1:100
BV605	CD4	RPA-T4	Biolegend	300556	1:200
BV650	CD3	UCHT1	Biolegend	300468	1:100
BV711	CD45RO	UCHL1	Biolegend	304236	1:100
BV785	CD95	DX2	Biolegend	305646	1:100
PE	CD154	5C8	Miltenyi	130-113-607	1:200
PE-Vio615	CXCR3	REA232	Miltenyi	130-118-967	1:100
PE-Vio770	CD38	IB6	Miltenyi	130-113-428	1:50
APC	CD69	FN50	Biolegend	310910	1:200
R718	CD45RA	HI100	BD Horizon	567073	1:500
APC-Cy7	CCR7	G043H7	Biolegend	353212	1:50
BUV395	CD2	RPA-2	BD Horizon	563819	1:25
BUV737	CD25	2A3	BD Horizon	564385	1:200
APC	mTrbc	H57-597	BD Biosiences	553174	1:50
PE	CD4	RPA-T4	Biolegend	300508	1:200

Validation

Antibodies were validated using flow cytometry of human PBMC and obtaining the correct pattern of staining vs other relevant markers and forward vs side scatter as shown in Figure S13. Antibodies were validated by vendors as follows.
Clone REA339 SLAMF6 Knockout validation of specificity, competition with other SLAMF6 antibodies, and testing against hybridoma clones. No information is provided for how human specificity was determined.
Clone SK1 CD8 specific for human CD8-alpha, references PMID: 21677135, Bernard A. A. Bernard .. et al., ed. Leucocyte typing : human leucocyte differentiation antigens detected by monoclonal antibodies : specification, classification, nomenclature.
Clone HIB19 CD19 specific staining of PBMC and B cell follicles in human spleen slices. References PMID: 36052346, PMID: 37928552
Clone 61D3 CD14 specific staining of PBMC in the monocyte gate by flow cytometry. Additional vendor specificity verification includes western blot, ELISA, IHC, IF, ICC, ChIP, and IP. References PMID: 2052920, PMID: 24139637, PMID: 3335025.
Clone B159 CD56 specific staining of of human PBMC. References PMID: 8920872, Schlossman SF. Stuart F. Schlossman .. et al., ed. Leucocyte typing V : white cell differentiation antigens : proceedings of the fifth international workshop and conference held in Boston, USA, 3-7 November, 1993. Oxford: Oxford University Press; 1995.
Clone 6B11 specific for TCR V-alpha24-J-alpha18 (iNKT cell) by flow cytometry of PBMC vs CD3. References PMID: 28932640, PMID: 36463279.

Clone HP-3G10 CD161 specific staining of human PBMC vs CD4 by flow cytometry. References PMID: 22058222, PMID: 26088389, PMID: 26917055.
Clone L291H4 CCR4 specific staining of human CD3 T cells from PBMC using flow cytometry, reactivity to CCR4 transfected cells. References PMID: 32239644, PMID: 36522365.
Clone 11A9 CCR6 specific staining of PBMC using flow cytometry (B cells, T cells, and DCs). References PMID: 9169459, PMID: 11696578, PMID: 9886385.
Clone RPA-T4 specific for CD4 staining of human CD3 T cells from PBMC using flow cytometry. The RPA-T4 antibody binds to the D1 domain of CD4 (CDR1 and CDR3 epitopes) and can block HIV gp120 binding and inhibit syncytia formation, as well as blocking of T cell activation. References PMID: 19773555, PMID: 32726801.
Clone UCHT1 specific for CD3 staining of lymphocytes from PBMC using flow cytometry. Clone UCHT1 induces functional activation of T cells. References PMID: 18490743, PMID: 32737409, PMID: 32497499.
Clone UCHL1 specific for CD45RO differentially stains human T cells vs CD45RA to identify memory T cells from PBMC using flow cytometry. References PMID: 18490743, PMID: 33789089, PMID: 29802019.
Clone DX2 specific for CD95 (Fas) staining of lymphocytes from PBMC using flow cytometry. The DX2 antibody is useful for inducing apoptosis of Fas-positive cells. References PMID: 20139273, PMID: 32610077, PMID: 34270918.
Clone 5C8 CD154 specific staining of stimulated CD4 T cells from PBMC but not unstimulated cells. Extended validation using epitope competition with other CD154 antibodies and staining of stimulated hybridoma clones. Reference PMID: 16186818.
Clone REA232 CXCR3 specific staining of CD3 T cells from PBMC compared to non-CD3+ cells by flow cytometry. Extended validation using epitope competition with other CXCR3 antibodies and staining of stimulated hybridoma clones. References PMID: 15150261, PMID: 21518913.
Clone IB6 CD38 specific staining of non-CD19 cells from PBMC compared to CD19+ cells. Extended validation using knockout validation for specificity and staining of stimulated hybridoma clones. Reference PMID: 20372106.
Clone FN50 CD69 specific staining on PMA and ionomycin stimulated PBMC by flow cytometry. References PMID: 18490743, PMID: 31229899, PMID: 32990219.
Clone HI100 specific for CD45RA differentially stains human T cells vs CD45RO to identify naiveT cells from PBMC using flow cytometry. References PMID: 22184723, Zola H. Leukocyte and stromal cell molecules : the CD markers. Hoboken, N.J.: Wiley-Liss; 2007.
Clone G043H7 CCR7 specific staining of CD3 T cells from PBMC by flow cytometry to identify naive, central memory, and effector memory T cells. References PMID: 35095881, PMID: 27870882, PMID: 27870882.
Clone RPA-2 CD2 specific staining on T cells and NK cells from PBMC by flow cytometry. References PMID: 2908548, PMID: 7681075, PMID: 1706002.
Clone 2A3 specifically binds to human CD25, the low-affinity alpha subunit of the Interleukin-2 Receptor (IL- 2Rα) on T cells from PBMC by flow cytometry. References PMID: 3930953, PMID: 3011033, PMID: 2403487, PMID: 12200381.
Clone H57-597 specifically binds to the murine Trbc protein on CD8+ Balb/C splenocytes compared to a negative control using flow cytometry. H57-597 has no reactivity to gd-TCR+ T cells. Plate bound H57-597 can activate murine T cells and induced apoptosis. References PMID: 7973703, PMID: 18432927.
Clone RPA-T4 specific for CD4 detects CD3+CD4+CD8- lyphocytes from PBMC using flow cytometry. RPA-T4 blocks HIV-gp120 binding to CD4 T cells and inhibits syncytia formation. References PMID: 35256819, PMID: 26553076.

Clinical data

Policy information about clinical studies
All manuscripts should comply with the ICMJE guidelines for publication of clinical research and a completed CONSORT checklist must be included with all submissions.

Clinical trial registration	Some samples were taken from placebo-treated subjects in the AbATE (NCT00129259) and START (NCT00515099) trials.
Study protocol	Study protocols and approvals are described in Supplemental Methods.
Data collection	Data were collected at multiple sites, as described in Table 1 and TableS1.<br><br>Cohort 1 samples: T1D and HC donors were from the Benaroya Research Institute Disease Registry and Repository (BRI DRR) and were collected in 2019-2021. newT1D samples were from the ITN T1DAL trial (2009-2014).<br><br>Cohort 2 samples: new T1D samples were from placebo-treated donors from the START (2007-2013) and AbATE (2005-2011) clinical trials. At risk samples were collected from TrialNet TN01 participants or BRI Diabetes registry participants in 2019-2021.<br><br>nPOD samples from pancreatic autopsy material was provided by Network for Pancreatic Organ Donors with Diabetes (nPOD) at: the ADI, Alberta Diabetes Institute Islet Core; VUMC/Pittsburgh, Vanderbilt University Medical Center/University of Pittsburgh; and the IIDP, Integrated Islet Distribution Program, and were collected in 2005-2022.
Outcomes	The primary endpoints of the T1DAL START and AbATE clinical trials were based on preservation of C-peptide area under the curve (AUC) following a mixed-meal tolerance test (MMTT) after 1 year.

Plants

Seed stocks	NA
Novel plant genotypes	NA
Authentication	NA

Flow Cytometry

Plots

Confirm that:

☒ The axis labels state the marker and fluorochrome used (e.g. CD4-FITC).

☒ The axis scales are clearly visible. Include numbers along axes only for bottom left plot of group (a 'group' is an analysis of identical markers).

☒ All plots are contour plots with outliers or pseudocolor plots.

☒ A numerical value for number of cells or percentage (with statistics) is provided.

Methodology

Sample preparation	Thawed PBMC were stained as described in a previous publication (PMID: 33351781).
Instrument	A BD FACSAria Fusion flow cytometer was used for cell sorts and acquisition, respectively.
Software	BD Diva software was used for cell sorts and FlowJo version 9.4 was used for analyses.
Cell population abundance	Cells were single cell sorted. Population abundance was determined from the parent gated population (CD4+CD154+CD69+)
Gating strategy	Gating is described in Supplemental Figure S13.

☒ Tick this box to confirm that a figure exemplifying the gating strategy is provided in the Supplementary Information.
